# Correction: A novel and actual mode for study of soil degradation and transportation of difenoconazole in a mango field

**DOI:** 10.1039/c9ra90048a

**Published:** 2019-06-20

**Authors:** Fangfang Zhao, Jingkun Liu, Defang Xie, Daizhu Lv, Jinhui Luo

**Affiliations:** a Analysis & Testing Center, Chinese Academy of Tropical Agricultural Sciences Haikou Hainan 571101 China luocatas@21cn.com; b Laboratory of Quality & Safety Risk Assessment for Tropical Products (Haikou), Ministry of Agriculture Haikou Hainan 571101 China; c Hainan Provincial Key Laboratory of Quality and Safety for Tropical Fruits and Vegetables Haikou Hainan 571101 China; d Quality Supervision and Inspection Center of Tropical Agro-Products Haikou Hainan 571101 China; e Environment and Plant Protection Institute, Chinese Academy of Tropical Agricultural Sciences Haikou Hainan 571101 China

## Abstract

Correction for ‘A novel and actual mode for study of soil degradation and transportation of difenoconazole in a mango field’ by Fangfang Zhao *et al.*, *RSC Adv.*, 2018, **8**, 8671–8677.

The authors regret that the author contributions were incorrect in the original article. Two co-first authors were originally indicated, however Fangfang Zhao should be the only first author as indicated here.

The Royal Society of Chemistry apologises for these errors and any consequent inconvenience to authors and readers.

## Supplementary Material

